# Living with dementia in a nursing home, as described by persons with dementia: a phenomenological hermeneutic study

**DOI:** 10.1186/s12913-017-2053-2

**Published:** 2017-01-31

**Authors:** Marit Mjørud, Knut Engedal, Janne Røsvik, Marit Kirkevold

**Affiliations:** 1 Norwegian National Advisory Unit on Ageing and Health, Vestfold Hospital Trust, Aldring og Helse, Postboks 2136, 3103 Tønsberg, Norway; 2 Department of Geriatric Medicine, Oslo University Hospital, Aldring og Helse, Postboks 2136, 3103 Tønsberg, Norway; 3 Department of Nursing Science, Institute of Health and Society, University of Oslo, Oslo, Norway

**Keywords:** Phenomenological, Interviews, Dementia, Nursing home, Quality of life

## Abstract

**Background:**

Persons with dementia have described life in nursing home as difficult and lonely. Persons with dementia often reside in nursing homes for several years; therefore, knowledge is needed about how quality of life is affected in the nursing-home setting in order to be able to provide the best possible care. The aim of this study was to investigate the personal experience of living in a nursing home over time from the perspective of the person with dementia and to learn what makes life better or worse in the nursing home.

**Methods:**

A phenomenological hermeneutic research design was applied. Unstructured, face-to-face interviews and field observations were conducted twice, three months apart.

**Results:**

Twelve persons residing in three different nursing homes were included. The analysis revealed four themes: “Being in the nursing home is okay, but you must take things as they are”; “Everything is gone”; “Things that make it better and things that make it worse”; and “Persons – for better or worse? Staff, family, and co-residents”.

**Conclusions:**

Persons with dementia are able to communicate their feelings and thoughts about their lives in the nursing home and can name several factors that have impacts on their quality of life. They differentiate between members of the staff, and they prefer their primary nurse. They are content with life in general, but everyday life is boring, and their sense of contentment is based on acceptance of certain facts of reality and their ability to adjust their expectations.

## Introduction

In Norway and internationally, a substantial number of the residents in any nursing home (NH) suffer from a dementia disorder. Approximately 28 000 persons live in NH in Norway. Of these, more than 80% have a dementia disorder [1]. World wide approximately 47.5 million people live with dementia [2]. To provide the best possible care, we need knowledge about how persons with dementia (PWD) experience life in the NH and how their quality of life (QOL) is affected by living in the NH.

## Background

The last decade has provided only a few qualitative studies focusing on life or QOL of PWD in NHs [3–5] through interviews with persons with dementia. The 81 care home residents included in the study by Clare et al. [6] all had moderate or severe dementia, and the data was collected through several conversations with the residents. The informants described living in a residential care home as a life of isolation, uncertainty and fear, and they tried to cope by accepting and making the best of things. The 61 NH residents included in the semi-structured, face-to-face interview study by Cahill et al., [3] suffered from mild, moderate, and severe dementia. The authors identified four key themes; social contact, attachment, pleasurable activities and affect. Particularly the persons with severe dementia told of an absence of social contact in the NH. Moyle et al. in Australia used semi-structured, face-to-face interviews to collect data from persons with mild to moderate dementia in long term care facilities [4, 5]. They studied loneliness and factors influencing on quality of life [4, 5]. Similar to the other studies, the results showed that social interactions and taking part in activities [4, 5] are important for the quality of life of persons with dementia.

By interviewing persons with dementia living in long-term care settings, other studies have explored specific foci such as unmet needs [7], awareness of self (in residential care homes) [8, 9], and activities (in care homes and nursing homes) [10–12]. The PWD describe how they maintain their identity and cope with and accept living in the NH by remembering their past [4, 6, 8, 9, 11], and they describe life as difficult [6, 13, 14]. They report feeling worthless and not being seen or heard as individuals [6, 13]. They also describe feeling homesick and feeling that they do not experience a sense of belonging in the NH [13], feeling captive, isolated [3, 6, 13], and lonely [3, 14].

To counter these feelings, NH staff members play an important role in helping the person with dementia in his or her everyday life [4, 10]. To have a positive effect, however, the interaction must be of “good quality” [11]; however, staff members have been found to be busy and unavailable [4], and PWD report that fixed environmental structures and activity schedules may result in boring and monotonous days with limited opportunities [4, 7, 10–12].

Furthermore, it is important for the PWD to partake in meaningful activities [5, 10–12]. What a meaningful activity entails, however, depends on each person’s preference, as it is the quality of the experience of the activity rather than a specific type of activity that is of importance [11]. Being able to perform activities independently [4], like choosing what to do and when to do it [11, 12], and engaging in social activities with family, friends [5, 11], and staff [4] have been found to have a positive effect on QOL in NHs [3–5, 11]. In addition, privacy and personal belongings are described by PWD to be important when living in a NH [4, 12, 13].

One Norwegian NH study has examined how dignity is preserved and described by PWD in NH [13] through interviews and field observations. One study has investigated thriving in NHs in cognitively intact persons [15]. Another has investigated life through field observations of the public spaces of the NH (i.e. the common living room, dining room, etc.) and individual interviews including cognitively intact persons [16].

As only a few studies have investigated life [6] and quality of life in general in the NH [3–5] and no studies have been conducted regarding this issue in Norwegian NHs, more research is needed. Furthermore, as only one study has used follow-up conversations with the PWD [6] and no studies have combined interviews with observations, the present study aims to use both in order to investigate the personal experience of living in a NH over time and what makes life better or worse from the perspective of the person with dementia using dovetailing sources.

## Method

A phenomenological hermeneutic research design was used inspired by Ricoeur [17] and further adjusted to empirical studies by Lindseth & Norberg [18]. This method can be used to study the essential meaning of the lived experience of a particular phenomenon through the interpretation of text. The essential meaning of a phenomena can be expressed trough narratives and through actions by the informant. Whereas phenomenology focuses on how meaning arises through subjective experience, hermeneutics highlights the necessary conditions for text interpretation [18–20].

In addition, dementia care mapping methodology [21] was used for the observations of everyday life in the nursing home ward and the observations were used as support mechanism for the interpretation of the interviews [22].

### Sample

PWD who had lived in a NH for a maximum of six months and with a life expectancy of more than three months were eligible for inclusion. The head nurse in the unit asked the persons with dementia and/or the relative if the person with dementia could not consent on his/her own, whether he/she would participate. All persons accepted participation. Because we wanted to include mostly persons with moderate and severe dementia, the severity of dementia was rated using the clinical dementia rating scale (CDR) [23]. The scale was filled in by the resident’s primary nurse. The primary nurses are usually nursing assistants with a vocational education. The CDR has six questions that focus on memory, orientation (time and place), problem solving, community affairs, home and hobbies, and personal care; each question has five possible answers. A score of 0 indicates no dementia, and a score of 3 indicates severe dementia. The person with dementia was also asked during the observation and interview whether or not he/she wanted to partake in the study. If he/she showed any sign of not wanting to participate (for instance walked away or became angry) the interview/observation was terminated. Twice, interviews were not conducted because the person with dementia did not want to be interviewed (see Table 1).

**Table 1:** Characteristics of the residents

Facility	Gender/Name	Age	Level of function at first encounter	CDR	Time in ward after admission to the nursing home	
					1. Interview	2. Interview
No 1 SCU	Female, Lisa	94	Severe aphasia, used walker	2	2 months	6 months
	Female, Marta	78	Moderate aphasia, walked without aid	2	2 months	No interview 6 months
	Female, Mina	83	Moderate aphasia, walked without aid	2	2 months	6 months
No 2 SCU	Female, Ella	82	Moderate aphasia, used high walker	3	5 months	Deceased
	Female, Betty	81	Moderate aphasia, walked without aid	2	5 months	8 months
	Female, Hanna	76	Severe aphasia, walked without aid	2	5 months No interview	8 months
RU	Female, Vera	92	Mild aphasia, walked without aid	1	5 months	8 months
	Female, Nelly	94	Severe aphasia, used high walker	2 (3)	5 months	8 months
No 3 RU	Male, Bob	71	Severe aphasia, used walker	2	4 months	7 months
	Male, Peter	83	Moderate aphasia, walked without aid	2	5 months	9 months
	Female, Mary	95	Moderate aphasia, used wheelchair	2	5 months	9 months
	Female, Anna	90	Mild aphasia, used walker	2	3 months	Ill – no interview

### Data collection

The interviews were organized around three topics:

- Thriving – contentment and a feeling of belonging
- Occupational or meaningful activities – activities provided in the nursing home and other activities
- Relationships – with staff, family, and other residents in the nursing home

The researcher (the first author), who is a registered nurse with clinical experience from nursing homes, met with each informant twice, approximately three months apart. Data were collected using face-to-face interviews and four hours of field observations at each meeting. The mood and engagement in activities of each person with dementia were documented in written notes during the observations using dementia care mapping methodology. Dementia care mapping uses five-minute time frames to map engagement in activities and mood, and interaction with other residents and staff [21]. The interviews had an unstructured form, and the participants were encouraged to talk freely about issues they thought were important in their lives in the NH. Questions were phrased in simple, everyday language. If the PWD did not understand the question, the question was rephrased. The questions were based in prior research literature on quality of life and theory on person centered care [24, 25]. All the interviews started with the researcher asking the participant what it felt like to live in the NH. If the participant denied that he or she was living in a NH, the question was rephrased to “here, where we are now”. Questions about whether they felt content, whether they thrived, and whether they felt safe in the setting were also asked during the interview. The term “thriving” was used to explore whether the physical and psychosocial frames in the NH gave the informants a positive subjective experience of life [26]. The rest of the questions were all follow-up questions based on the answers from the informants. However, if the PWD did not touch upon topics the researcher assumed relevant for life in the NH, they were asked questions about these topics, such as activities (i.e. How do you like the activities here? Are there activities that you miss? Are there some things you would like to do?); the staff (How are the staff here? Does the staff treat you well? Are there some staff members you like better than others?); and family (Does your family come to visit?) and co-residents (Do you have friends among the other residents?). The topic of personal belongings was not part of the initial interview guide but was included as it appeared to be important for two of the first three participants to show the first author items they had made and to talk about these. However, most participants talked about this without being asked.

### The data analysis

The interviews were audio-recorded and transcribed verbatim. All but three interviews were transcribed by the first author. The last three interviews were transcribed by a consultant at the Norwegian National Advisory Unit for Ageing and Health, and were subsequently read while listening to the interviews in order to ensure that the transcriptions were correct.

The analysis of the interviews is based in text analysis as described by Ricoeur [17, 18, 27] with naïve interpretation, structured analysis, and comprehensive understanding. The observations were not analyzed on their own but rather as a support mechanism for the interpretation of the interviews because of the residents’ cognitive impairment and speech problems [22]. First, the entire content in the interviews was read for an overall impression about living in NH with dementia (naïve interpretation). Then, in the structured analysis, we conducted critical reading, which means that the text is broken down to meaningful units, and these units were compared to the observations. An example is the resident sleeping through an activity (i.e. quiz) and, during the interview, saying that the activities in the NH are boring. By reading the interviews critically, one gains a deeper understanding and discovers similarities in the content across the interviews. An example is several residents talking positively about the primary nurse, and the observations confirming that the primary nurse is the one who most often talks to the resident during the observation. The units are gathered in themes or characteristics of living in the NH. After reflecting upon the themes, the interviews were reread to determine whether the emerged units gave a new understanding of the material. The final themes were reflected upon in relation to the research question and the context of the study (comprehensive understanding) [18], and the credibility of the final interpretation was secured through grounding of the meaningful entities in the text through phrases from the respondents [27, 28] and by comparing the phrases with observations made in the unit. An example is the quote “the staff is annoying”, which was observed as staff moved wheelchairs without first telling the residents. The analysis was done by the first author in cooperation with the co-authors.

### Ethics

The study was approved by the Regional Committee for Medical and Health Research Ethics in Southeast Norway using the following procedure: Participants were informed in writing as well as verbally. Competency was evaluated by the nurse in charge in the nursing home, and when competent, the PWD was asked to give his or her informed consent. If not competent, the next of kin was asked to approve or reject participation [29]. In addition, the PWD was informed about the study and asked whether she or he wanted to participate before and/or during each interview [30, 31]. Furthermore, the interview with the PWD was terminated if he or she physically indicated that he or she did not wish to continue, for instance, if the PWD appeared to be nervous or agitated, or walked away. Pseudonyms have been used to preserve anonymity of the participants.

## Results

### Participants

Data saturation was reached after we had included 12 PWD, 10 (83%) women and two (17%) men, ages 71–95 years, residing in three different NHs. Six lived in special care units (SCU) for persons with dementia, and six lived in regular units (RU). In the SCU only persons with dementia reside. Most persons with dementia in SCU have some kind of challenging behavior. In the RU people who no longer can live at home due to any kind of disease, including dementia with no serious behavioral problems reside. In Norway the staffing is slightly higher in the SCU compared to RU, with a staff ration of 0.3 staff per patient in RU, and 0.35 in SCU [32].

The men lived in the same NH but in separate units. Characteristics of the residents can be seen in Table 1. One person died before the follow-up interview; one did not want to be interviewed the first time but consented the second time; one did not want to be interviewed the second time; and one person became severely ill and could not participate in the second interview, for a total of 20 interviews.

The primary nurse is usually an vocational nurse or in some cases a registered nurse, who has got special responsibility to take care of the person with dementia, being in touch with the family, making sure the room is tidy, that the person with dementia has everything he/she needs etc.

Quotes are marked ^1^ and ^2^ respectively, indicating the interview from which they are taken.

In the 20 interviews, no differences were found with regard to the topics that the PWD talked about. However, the residents had, in general, become more resigned from the first to the second encounter. An example is Betty, who at the first interview said, *“I hope it (life) will be a bit longer”*,^*1*^ and during the second interview said, “*Now it is over.(…)… Let’s hope it’s not much more”.* ^*2*^ Still, she talked about the same topics, that is, her earlier life with her husband and children, and how it is necessary to have a positive attitude toward life: *“[if you can’t be home], life can’t be [expected to be] better than this”.* ^*2*^

### Themes

Four main topics emerged from the analysis:

1. “Being in the nursing home is okay, but you must take things as they are”
2. “Everything is gone”
3. “Things that make it better and things that make it worse”
4. “People – for better or worse? Staff, family, and co-residents”

#### Being in the nursing home is okay, but you must take things as they are

Many of the residents seemed to have a dissolved understanding of time, and they did not necessarily have an understanding of where they were or why they were in the nursing home. Nevertheless, most of them expressed contentedness about being in the nursing home: *“…as long as you are not home, it cannot be better than this. (…) if you come to a place, you must put some [effort] into it, [and put some things] behind you. I live here, I will be content here”.* ^*(Betty)1*^ Hanna said, *“…I am completely very good”* ^*2*^ indicating that she was content in the NH.

Regarding why they were living in the NH, most of the PWD perceived it as necessary: *“[I] need to be in the hospital…”.* ^(Mary) *2*^ They understood that they could no longer live at home alone; as Betty said, *“It is just that you are not strong enough to live at a farm…and do everything that needs to be done”.* ^*2*^ Although recognizing that they could not live in their own homes, several of the participants saw living in the NH as a temporary solution: *“…it is alright to be here for an evening, a year or a year… so I will not…hurry it or something like that…but when…when… finished I want to go home to her (his wife)”.* ^*(Peter) 1*^And Mina said*, “I think it is alright, but not all the time, no I don’t want that”.* ^*1*^ Mary explained why: “*[*…*because it is] a heavy burden [being here]”.* ^*2*^

None of the residents regarded the NH as a real home. They missed their old homes and wished they could be there. Mina said, *“Yes, I would rather be at home, but I probably couldn’t. I am so old (crying) that I couldn’t do anything anymore”.* ^*1*^ She continues to see the NH not as a home, but during the second interview she says, *“It is not home, but I go around dancing and enjoying myself. I do that because I must live my life…”.* ^*2*^

They tried to make the best of the situation, accepting that they had to follow the routines in the NH: *“You cannot sit…and [expect that] it should be like this this this…”.* ^*(Betty) 1*^ Still, they found it difficult that the days were much alike, as Betty said, *“…and it is difficult because it is much the same that recurs”.* ^*2*^ And Mary said, *“No…I…I don’t think it is…it is tempting to…to live… there…I think…it is…much…the same…same”.* ^*1*^

They talked about adjustment of expectations. Betty explained*, “If you can’t be home, you must be happy you are on your feet and can have your own room”.* ^*2*^ Vera talked about how her expectations came true: *“It is…you know how it is, in a nursing home”.* ^*2*^ They described how they must accept how things are done, as Marta explained, *“Yes, I think I like it here…but you cannot maybe…what should I say…pick and ask sort of.... You must take things as they are and just think, no, I must…”.* ^*1*^

#### Everything is gone

Most of the residents talked about their past lives and described a feeling of having lost those times. They emphasized the loss of family and home, and some also described the loss of self.

The residents described that, after a long life of working and saving, everything was now gone. Marta said, *“I have worked and made so many things and helped…. Gone”.* ^*1*^ And Vera expressed: *“All I have…saved through many years, bought and strived and worked for…it all have to go.. weird….”* ^*1*^ *.*

The residents described a feeling of having lost interest in doing things: *“I have lost the glow and all such sorts”,* ^(Vera) 2^ and also their capabilities: *“I can’t do anything anymore”.* ^(Nelly) 2^

A few of the residents described losing themselves, like Mina who did not recognize herself: *“I don’t know, but it is not me anyway. I like to do so much, and suddenly I am an old hag”.* ^*1*^ Some described themselves as worthless, like Lisa, who struggled to speak and described herself as rubbish: *“old…so… rubbish…”,* ^*1*^ and Nelly, who said, “*What can I do? Nothing”* ^1^ and *“I am good for nothing”.* ^2^

Several of the residents, particularly those with severe dementia, expressed feelings of loneliness. Lisa said, *“…I am…old…”* and *“I am so…lonely…”.* ^*1*^ And Ella, struggling to speak, said she was lonely,*“…especially during the evenings”.* ^*1*^

Most of the residents missed people in their families. They expressed feelings of love, as Peter said, *“I…love them”,* ^*2*^ and longing, as Lisa said, *“I miss my sister”.* ^*1*^ Mary said, *“My family… always in my thoughts”.* ^*2*^

Four of the residents felt that their families had left them in the NH and didn’t visit them often enough, like Mary, who said, *“…yes…so… I think that…always it is…my family…in my thoughts…and…both…and all…busy…I am…must be…busy busy…busy and happy that I am in neighboring country…”.* ^*2*^

#### Things that make it better and things that make it worse

The residents described people, belongings, and activities that made life in the NH better or worse.

As they were dependent on help for most of the activities of daily living (ADL), like dressing and bathing, the assurance that they would get help gave them a feeling of safety: *“I feel safe. Someone looks after you”.* ^*(Anna)1*^ Peter said, *“Yes, I do feel safe, there hasn’t been any frights”.* ^*2*^ In addition, the presence of medical personnel if they fell or became ill gave them comfort. During the observations, it was observed that the presence of the primary nurse made most of the residents smile.

Creating a personal space in the NH by having their own rooms with their personal belongings emerged as important for making things better: *“It’s to make it more homely”.* ^*(Betty)1*^ Mary, who had many hats, said, *“Yes…when it comes to hats…I do love…and I think…it is more…fun… fun…with hats”.* ^*1*^ Betty kept a picture of her husband to feel less lonely: *“It is sort of so you don’t feel so lonely…but that he is close”.* ^*1*^

However, some of the residents described being scared of losing things. Peter, for example, said, *“You can risk losing it…(.)…things happen slightly skewed and stuff…”.* ^*2*^

Belongings were used for reminiscence, but the memories of what used to be caused both happy and sad thoughts. Some used happy memories for comfort in the present situation, like Betty: *“I have decided to be very happy about what I had, very, very happy…and family and friends… because it is not coming back”.* ^*2*^ Others became sad, like Vera: *“It has been good…(…)…this I had never believed…never never never… no…that I would sit like this”.* ^*2*^

During several of the interviews, staff entered the residents’ rooms without knocking. This violation of their private space was obviously something the residents did not like. As Marta said, *“She is supposed to help us, and that there, that was simply rude”.* ^*1*^

Even though all three nursing homes had activity plans, the residents experienced most of the activities as boring. Typical statements were: *“Yes [it is boring]”* ^*(Vera)1, (Mary)1*^and *“It is quiet like the grave here”* ^*(Vera)2*^ and *“I have no interest in anything here”.* ^*(Anna)1*^ The observations confirmed the statements, as the residents slept through activities provided by the staff, typically quizzes or remembering proverbs. However, a few residents talked about music sessions they enjoyed, and this was also confirmed during the observations when the residents joined in singing. Peter wanted to attend church services when they were offered. No other participants mentioned activities provided by the NH that they wanted to attend.

However, several talked about past hobbies they missed, even if they did not necessarily think they could manage to do them: *“…You have to accept the situation, so…to do [the hobby]…no…”.* ^*(Betty) 2*^ Anna and Vera talked about activities they wanted to do that would make life better, like going outdoors or going to the shopping mall.

A few talked about being independent and that being able to dress, wash up, and tidy their room were important in order to feel content. Vera said, *“I want to be independent and…”*and *“…I use my head…as long as it lasts”.* ^*2*^

#### People – for better or worse? Staff, family, and co-residents

##### Staff

The staff emerged as an important part of the residents’ life in the NH. The residents described them as “*kind and friendly”* ^*(Nelly) 1*^, but challenges were also described: *“They are…very nice… but other times…then…more…annoying”* ^*(Mary)1*^ . An observed example of *annoying* conduct was for instance the staff moving wheel chairs without first telling the person sitting in the chair.

Not being alone was described as important, and Vera described how the staff would be there if she needed them: *“Can go to them and talk about everything… then I feel safe…”.* ^*1*^ Anna described how being together with other people made her feel:*“… I felt safe you know, it is better to be together with others…”.* ^*1*^ However, they also described how there are things they cannot talk about, as Peter said, *“I will never discuss it with him”* ^*1*^ and *“It is limited, what you can talk about”.* ^*(Peter) 2*^

The residents preferred to be helped by a few, familiar nurses, and especially the primary nurse, *“yes, prefer her”.* ^*(Bob)1*^ It was easy to see and understand that Bob preferred the primary nurse. During the first day of observation, she was the only one who approached him and talked to him. Peter said, *“It is often new…the sorts…consultants…nurses…yes…. It is just like something has torn.... I think you can say he* [the primary nurse] *is the one I like the best”.* ^*2*^

However, the residents described having to behave in certain ways and having to accept how things were done. Lisa said, *“Yes, they are nice…but we must be nice too”* ^*2*^ and *“Yes and you have to be…I have to put up with…”.* ^*(Lisa)2*^

Some residents described staff that did not meet or treat them in a way they approved. Marta described how she *“got the feeling I am just a brat she has to take care of”* ^*1*^ and how she felt about the staff: *“I can’t tell her I think she behaves rude. You see, she feels very important now, but she is only here to help”.* ^*1*^ Peter said this about his primary nurse, *“His hands are so strong too…and he squeezes a bit too hard…he still got some of that…he wants…I understand he wants me to remember…(…) I don’t have to scream but he understands I think he is too hard-handed…”.* ^*1*^

The residents experienced the staff as absent and that they often disappeared: *“He runs off, you see”.* ^*(Peter)1*^ When asked, *“Is it easy to find the staff?”,* Hanna, who had hardly any verbal language at all, replied, *“No, it is not easy, absolutely not”.* ^2^ The observations confirmed this, as the residents often sat alone in the living rooms or corridor.

##### Family

Having family members who visited them emerged as important to the residents’ lives: *“To get a hug when he leaves and a kiss when he leaves again…then the day is saved”* ^*(Betty)1*^ and *“It is good to have someone you can talk to”.* ^*2*^ The residents missed family members and looked forward to visits: *“She brings those… a couple of those…half-grown (grandchildren)…comes together with them…and she brings chocolate…”.* ^*(Peter)1*^

##### Other residents

Other residents were not an important topic to the PWD. Two women mentioned other residents whom they looked after, as Mina explained, *“…I could help them, and I do, if they want to, but many are in pain…hardly walk…”.* ^*1*^ Vera described the other residents this way: *“…They just sleep… just sit there and cry and sleep and cry…”.* ^*2*^

## Discussion

This study aimed to describe life in NHs for persons with dementia using the words of the residents and to investigate the factors that make life better or worse from their perspective. Four themes emerged.

Through the first theme, “Being in the nursing home is okay, but you must take things as they are”, the informants of the present study, in line with other studies [4–6], expressed that they were content being in the NH. However, all of the informants explained that their contentment was based on their acceptance of certain facts of reality. First, that they had to live there because they were not able to care for themselves any longer in their own home, acknowledging the positive sides to living in the NH. Second, that this was a place that required an adjustment in their expectations and compliance to routines, and that it did not provide the comfort of a home. None regarded the NH as a home and approximately half of the informants regarded their NH placement as a temporary solution [3, 6]. One way of interpreting this is that persons with dementia, similarly to persons without dementia [15], have the insight that the situation (of living in a NH) is necessary although not preferred, and they are able to adjust their expectations to what they see as realistic.

Through the second theme, “Everything is gone”, the informants talked about losing the important things that had earlier made up their life, like spending time with family and friends, having a home and a job to go to, and being interested in and able to pursue hobbies [6]. They missed their family members. Several other studies have found that visits from family members are important for QOL of PWD in NHs, even when the resident does not seem to know the visitor or forgets about the visit shortly after [3, 4, 6].

A few of the informants described not recognizing themselves. This has been described in several studies [5, 6, 9] and is a frightening feeling that could potentially lead to an increased level of depression and feelings of isolation. The nurses ought to receive guidance in how to practically use knowledge of the person with dementia’s life and his/her likes and dislikes to tailor the care they provide, to help the person with dementia continue to recognize him/her self.

The third theme, “Things that make it better and things that make it worse”, underscored that persons with dementia are able to name several factors that may have positive or negative impacts on their lives. Creating a personal space by having their own belongings and talking about their past lives were important for the residents. It seemed that belongings and photographs helped them to remember happier times in their lives and thus aided them in accepting and coping with their situations.

Having things to do has been found to be important for relieving loneliness [14] and for QOL of PWD in NHs [10]. In the present study, the residents enjoyed some of the activities provided in the NH, but mostly they found the activities to be boring and the days monotonous [10, 11]. Earlier studies have identified organizational issues as the main reason for this [7, 11, 33], including insufficient staff competence and poor attitudes, and the prioritization of physical needs over psychosocial needs. The results of the present study could indicate the same situation, as the residents often fell asleep during the activities, and they sat alone for large parts of the day, but this is unclear. It is of clinical importance to relieve loneliness to provide activities tailored to the interests of each resident would be beneficial for PWD, as also found in other studies [3, 11, 14].

The fourth theme, “People – for better or worse? Staff, family, and co-residents” underscored the PWD’s preferences for being surrounded by people they know, and in particular, the primary nurse emerged as an important person in the NH [3] in addition to family members. It is surprising that the PWD so clearly differentiated between the members of staff and recognized the primary nurse as important, in spite of the fact that the primary nurse did not always act in a respectful manner toward the PWD. In general, staff were described as friendly and kind, safeguarding the residents’ physical needs [13], but also as absent, annoying, and disrespectful. Physical absence and disrespectful behavior could be a result of a lack of understanding and knowledge about what a person with dementia actually perceives in regard to what is happening around her or him.

As in Cahill’s study, interactions between the residents were rare in our study [3], and the informants did not express feelings of friendship or companionship with the other residents.

## Strengths and limitations

Strengths of the study include the use of field observations in addition to interviews; few other studies have done this. As cognitive impairment might change the experience of his or her own body, life, and world, in addition to making communication difficult for the person with dementia, observing the PWD in everyday activities can help the researcher understand how he or she experiences life in the NH. However, a relatively short period of time was spent with the residents, approximately 8 to 10 h, and additional time would have been more beneficial.

The presence of aphasia could potentially be a limitation due to misunderstanding and difficult communication. However, the interviews revealed that the PWD could talk about his or her experience of living in a nursing home even with cognitive impairment, and the observations were consistent with and supported the interpretation of the statements they made.

A limitation is that the informants shared a similar background; that is, they were all ethnic Norwegians of about the same age. Including persons with different ethnic backgrounds could have given other results.

Another limitation is of course the fact that the head nurse was responsible for including the informants. The informants could perhaps have felt they had to participate since it was the head nurse who asked.

## Clinical implications

The NH staff members need knowledge about the important role they play regarding the everyday life and well-being of the PWD. The results from this study could be used to teach the care staff about this important role, and particularly, the role of the primary nurse should be strengthened through guidance and education. A positive relationship between the resident and the primary nurse might enhance the potential for a good life in the NH and reduce residents’ feelings of loneliness and depression. If the primary nurse has knowledge of a resident’s likes and dislikes, and knows how to use this knowledge practically, it is easier to tailor activities to the interests and needs of the resident [25, 34–36]. The clear statement from the PWD that life in the NH is boring calls for action from leaders in NHs and municipalities regarding what tasks the health care staff should be responsible for, for example whether they should do common house hold chores like cleaning instead of spending time with the resident, and whether more guidance and training in dementia care would be beneficial. The persons with dementia also found it difficult to find the care staff. For the care staff to be able to spend more time with and provide activities the residents like, the staff should get the opportunity to regularly discuss how to organize the day so they are available to the residents and are able to provide tailored activities.

As the persons with dementia express missing their family members, the care staff should also help family members by facilitating meaningful activities the family could do together with the person with dementia, so they find the visits more rewarding and maybe visit more often.

Another important issue that should be given increased attention in clinical practice and health care education is the ability of persons with dementia to perceive what is happening around them and their ability to verbally address this in spite of aphasia.

## Conclusion

This study demonstrates that persons with dementia are able to communicate their feelings and thoughts about life in NHs and to name several factors that have impacts on their lives in the NH. The results show that PWD differentiate between members of staff and that they prefer the primary nurse. The staff is mostly nice but also absent and sometimes disrespectful. PWD say that they are content with life in the NH but that it is boring. Their contentment is based on their acceptance of certain facts of reality and their ability to adjust their expectations.
